# Protective immunity induced by oral vaccination with a recombinant *Lactococcus lactis* vaccine against H5Nx in chickens

**DOI:** 10.1186/s12917-021-03109-z

**Published:** 2022-01-03

**Authors:** Yi Ren, Xin Lu, Zhonghe Yang, Han Lei

**Affiliations:** College of Medicine, Southwest Jiaotong University, Chengdu, 610031 Sichuan China

**Keywords:** *L. lactis*/pNZ8149-HA1-M2, HA1 and conserved M2, Protection, H5Nx virus infection

## Abstract

**Background:**

The development of an influenza vaccine for poultry that provides broadly protective immunity against influenza H5Nx viruses is a challenging goal.

**Results:**

*Lactococcus lactis* (*L. lactis*)/pNZ8149-HA1-M2 expressing hemagglutinin-1 (HA1) of A/chicken/Vietnam/NCVD-15A59/2015 (H5N6) and the conserved M2 gene of A/Vietnam/1203/2004 (H5N1) was generated. *L. lactis*/pNZ8149-HA1-M2 could induce significant humoral, mucosal and cell-mediated immune responses, as well as neutralization antibodies. Importantly, *L. lactis*/pNZ8149-HA1-M2 could prevent disease symptoms without significant weight loss and confer protective immunity in a chicken model against lethal challenge with divergent influenza H5Nx viruses, including H5N6 and H5N1.

**Conclusions:**

*L. lactis*/pNZ8149-HA1-M2 can serve as a promising vaccine candidate in poultry industry for providing protection against H5Nx virus infection in the field application.

**Supplementary Information:**

The online version contains supplementary material available at 10.1186/s12917-021-03109-z.

## Background

Highly pathogenic avian influenza viruses H5Nx are a severe threat to poultry health [1], as they can introduce mutations in the antigenic sites of the surface glycoprotein antigens hemagglutinin (HA) and neuraminidase (NA) [2]. The currently licensed inactivated influenza virus vaccines for poultry are whole viruses or disrupted viral antigens containing hemagglutinin and neuraminidase glycoproteins, which are the major targets of neutralizing antibodies [3, 4]. However, these vaccines that are based on neutralizing antibody responses to the highly variable influenza HA protein provide protection against only homologous but not antigenically distinct heterologous viruses [5]. Therefore, efforts are being undertaken to develop a safe and effective vaccine for poultry that would be able to induce protective immunity against divergent influenza H5Nx viruses.

Influenza A virus contains a highly conserved ectodomain of matrix protein 2 (M2e) exposed on the surface of the virion, a particularly attractive target for the development of vaccines that induce broad protection [6]. Despite the presence of the invariant domain of M2, antibody responses to M2 are insufficient after vaccination, indicating that M2 is poorly immunogenic [7]. To enhance the immunogenicity of M2e, some strategies have been reported for immunization with M2e peptide fusion constructs linked to carrier vehicles as vaccine candidates, which have been shown to have safety issues but provide protection against lethal infection in chickens [8, 9]. However, these M2e vaccine candidates provide limited protection against lethal challenge, as shown by significant weight loss and signs of disease, even in the presence of potent adjuvants, such as heat-labile endotoxin or cholera toxin, Freund adjuvants, or bacterial protein conjugates [10, 11]. These previous studies further suggest that the immunogenicity of M2 linked to carrier vehicles may not be sufficient to prevent viral infection, and the adjuvant agents may not be approved for use in poultry as a result of their potential adverse effects. Thus, there is a clear need for new vaccine formulation and delivery strategies that can provide increased efficacy and safety.

Several different vaccination strategies for chickens via the non-invasive methods have been developed against avian influenza viruses including replication-deficient adenovirus viral vector [12], Newcastle Disease Virus (NCD) [13], recombinant fowl-pox virus (FPV) [14], nanoparticle [15] as well as virus-like particles (VLPs) [16]. Therefore, mucosal delivery has exhibited the potential of avian influenza vaccine for poultry.

*L. lactis* is an attractive choice and a safer mucosal vaccination strategy against pathogens. Since the safety profile of *L. lactis* is well established, this organism has significant appeal as a mucosal vaccine delivery vector [17, 18]. Various heterologous bacterial and viral antigens have been expressed on *L. lactis*, and antigen-specific immune responses have been reported [19, 20]. Notably, recombinant *L. lactis* has been engineered for the design and development of influenza vaccines [21–23]. The results of our previous studies have shown that oral vaccination with recombinant *L. lactis* expressing the NA or HA gene of the H5N1 virus could induce influenza virus-neutralizing antibodies and provide protection against H5N1 infection in chickens [21, 22]. Furthermore, intragastric delivery of recombinant *L. lactis* expressing influenza NA or M2e proteins could induce effective mucosal and systemic immune responses and protect MDCK cells against avian influenza type A/PR/8/34 (H1N1) virus challenge [24]. However, little is known regarding whether *L. lactis* based vaccines can provide protective immunity in poultry against divergent influenza H5Nx viruses.

Influenza viral HA is a receptor-binding protein that consists of a globular head (HA1) and a stem domain (HA2). Because the HA1 subunit has strong immunogenicity and predominantly induces neutralizing antibodies [3], while M2 has cross-reactivity [25], we hypothesized that food-grade recombinant *L. lactis* expressing HA1-M2 (*L. lactis*/pNZ8149-HA1-M2) would elicit strong and protective immunity against divergent influenza A types in a chicken model. The results of the present study demonstrated that *L. lactis* based vaccine can be considered an effective platform for an influenza vaccine production for mass vaccination in poultry, providing protective immunity in the absence of a mucosal adjuvant during H5Nx outbreaks.

## Results

### Expression of the HA1-M2 fusion protein in *L. lactis*

**Fig. 1:**
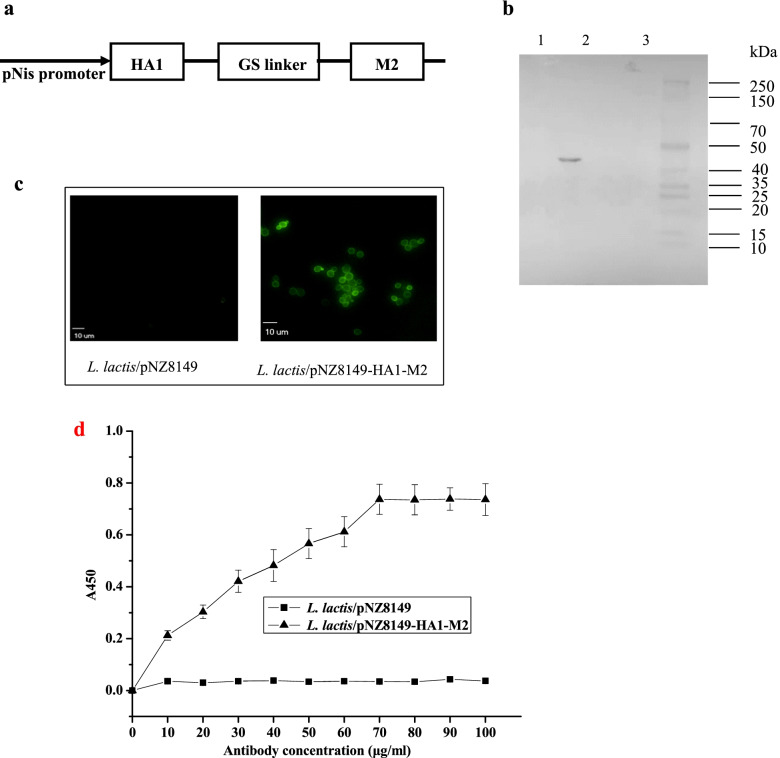
Expression of the HA1-M2 fusion protein in *L. lactis*. **a** Schematic diagram of *L. lactis*/pNZ8149-HA1-M2. **b** Western blot analysis. M: Western blot marker; Lane 1: Cell culture supernatants of *L. lactis*/pNZ8149-HA1-M2; Lane 2: Cell lysates of *L. lactis*/pNZ8149-HA1-M2 (approximately 45 kDa); Lane 3: Cell lysates of *L. lactis*/pNZ8149; Lane 4: Cell culture supernatants of *L. lactis*/pNZ8149. **c** Immunofluorescence microscopy assay. *L. lactis*/pNZ8149 (left) and *L. lactis*/pNZ8149-HA1-M2 (right) (magnification: 1000×). d Quantification of *L. lactis*/pNZ8149-HA1-M2 expressing the HA1-M2 fusion protein measured by whole-cell ELISA. A450 values were obtained from three independent experiments. The bar indicates the means ± SDs

To confirm the expression of the HA1-M2 fusion protein, Western blot analyses of cell extracts and cell supernatants were performed. No bands were observed in either *L. lactis*/pNZ8149 cells or their culture medium (Fig. 1b, Lane 3 and Lane 4). Whereas an immunologically specific protein band of the expected size was detected only in the *L. lactis*/pNZ8149-HA1-M2 cells with a molecular mass of 45 kDa (Fig. 1b, Lane 2), no bands were detected in the *L. lactis*/pNZ8149-HA1-M2 cell supernatants (Fig. 1b**,** Lane 1). In summary, the expressed proteins were located exclusively in the cytoplasm of *L. lactis*/pNZ8149-HA1-M2 cells.

To evaluate the intracellular expression of the HA1-M2 fusion protein, *L. lactis*/pNZ8149 or *L. lactis*/pNZ8149-HA1-M2 cells were probed with a monoclonal mouse anti-HA or anti-M2 antibody and then analyzed by immunofluorescence assay. A consistently high expression level of the HA1-M2 protein was observed on the *L. lactis*/pNZ8149-HA1-M2 cells (Fig. 1c**,** right panel), and no specific fluorescence signals were detected on the *L. lactis*/pNZ8149 cells (Fig. 1c, left panel).

The amount of *L. lactis*/pNZ8149-HA1-M2 expressing the HA1-M2 fusion protein was determined by whole-cell ELIA. As shown in Fig. 1d, the absorbance value (A450) of 1 × 10^8^ CFU *L. lactis*/pNZ8149-HA1-M2/100 μl was relatively stable, when the concentration of antibodies was increased at 70 μg/ml. Therefore, 1 × 10^8^ CFU *L. lactis*/pNZ8149-HA1-M2 expressed 7 μg (80 μg/ml × 100 μl = 7 μg) of the HA1-M2 fusion protein.

### Humoral and mucosal immune responses induced by *L. lactis*/pNZ8149-HA1-M2

**Fig. 2:**
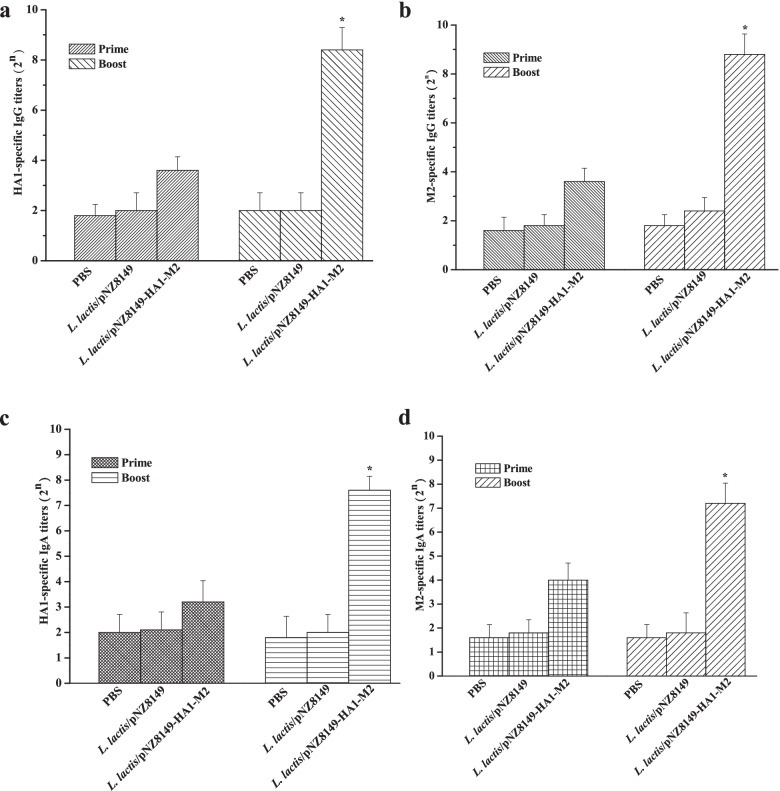
Humoral and mucosal immune responses elicited by *L. lactis*/pNZ8149-HA1-M2 in chickens. Sera and the intestine washes were collected from chickens vaccinated orally with PBS, *L. lactis*/pNZ8149 or *L. lactis*/pNZ8149-HA1-M2 at day 14 and day 28 after the first immunization. **a** HA1-specific IgG antibody responses in the sera (*n* = 10 chickens/group). **b** M2-specific IgG antibody responses in the sera (*n* = 10 chickens/group). **c** HA1-specific IgA antibody responses in the intestine washes (*n* = 5 chickens/group). **d** M2-specific IgA antibody responses in the intestine washes (*n* = 5 chickens/group). Data are represented as the mean ± standard deviation (SD). Asterisks indicate significant differences compared to the PBS and *L. lactis*/pNZ8149 controls (*p* < 0.05)

To investigate the humoral immune responses induced by *L. lactis*/pNZ8149-HA1-M2, serum HA1- or M2-specific IgG titers were determined by ELISA. No significant serum IgG antibodies were detected in any group at day 14 after the first immunization. However, HA1- or M2-specific IgG antibodies were significantly induced by *L. lactis*/pNZ8149-HA1-M2 at day 28 (Fig. 2a, b). In contrast, a low HA1- or M2-specific IgG titer was shown in the PBS or *L. lactis*/pNZ8149 group after the boost immunization (Fig. 2a, b**).**

Furthermore, HA1- or M2-specific secretory IgA titers were also determined in the intestine washes collected from the vaccinated chickens. As shown in Fig. 2c and d, there were no detectable IgA levels in all groups after the prime immunization (at day 14) since all of IgA titers were less than 2^4^, which was considered the background level. Only *L. lactis*/pNZ8149-HA1-M2 induced a higher HA1- or M2-specific IgA antibody response after the boost immunization (at day 28).

Taken together, these results demonstrate that *L. lactis*/pNZ8149-HA1-M2 is strongly immunogenic after prime-boost immunization that was able to elicit significant humoral immune responses, as well as mucosal immune responses, which may correlate to preventing viral infection.

### Cellular immune responses elicited by *L. lactis*/pNZ8149-HA1-M2

**Fig. 3:**
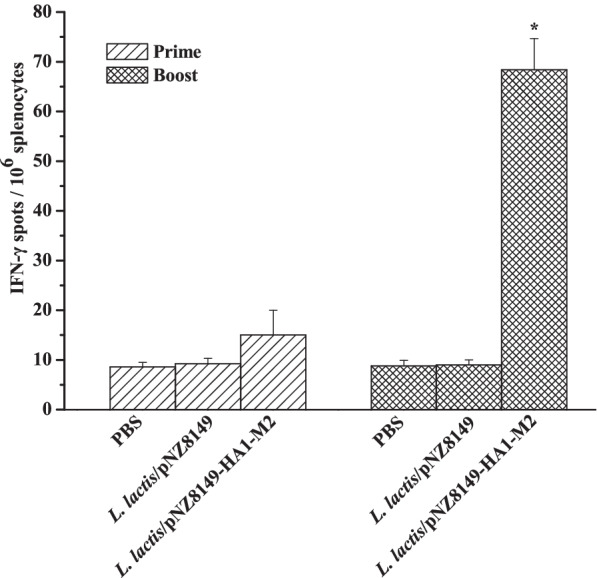
Cellular immune responses induced by *L. lactis*/pNZ8149-HA1-M2. Less than 20 IFN-γ spots were considered not statistically significant (*n* = 5 chickens/group). Data are represented as the mean ± SD. Asterisks indicate significant differences compared to the PBS and *L. lactis*/pNZ8149 controls (*p* < 0.05)

To test the cellular immune responses elicited by *L. lactis*/pNZ8149-HA1-M2, IFN-γ-secreting splenocytes were determined by ELISpot assay according to the manufacturer’s protocol. As shown in Fig. 3, significant numbers of IFN-γ spots were detected in *L. lactis*/pNZ8149-HA1-M2 group at day 14 after the first immunization and reached the highest level at day 28. However, there were no detectable IFN-γ spots in the PBS or *L. lactis*/pNZ8149 group. Overall, these results demonstrate that *L. lactis*/pNZ8149-HA1-M2 can elicit strong cellular immune responses.

### HI assay and microneutralization assay

**Fig. 4:**
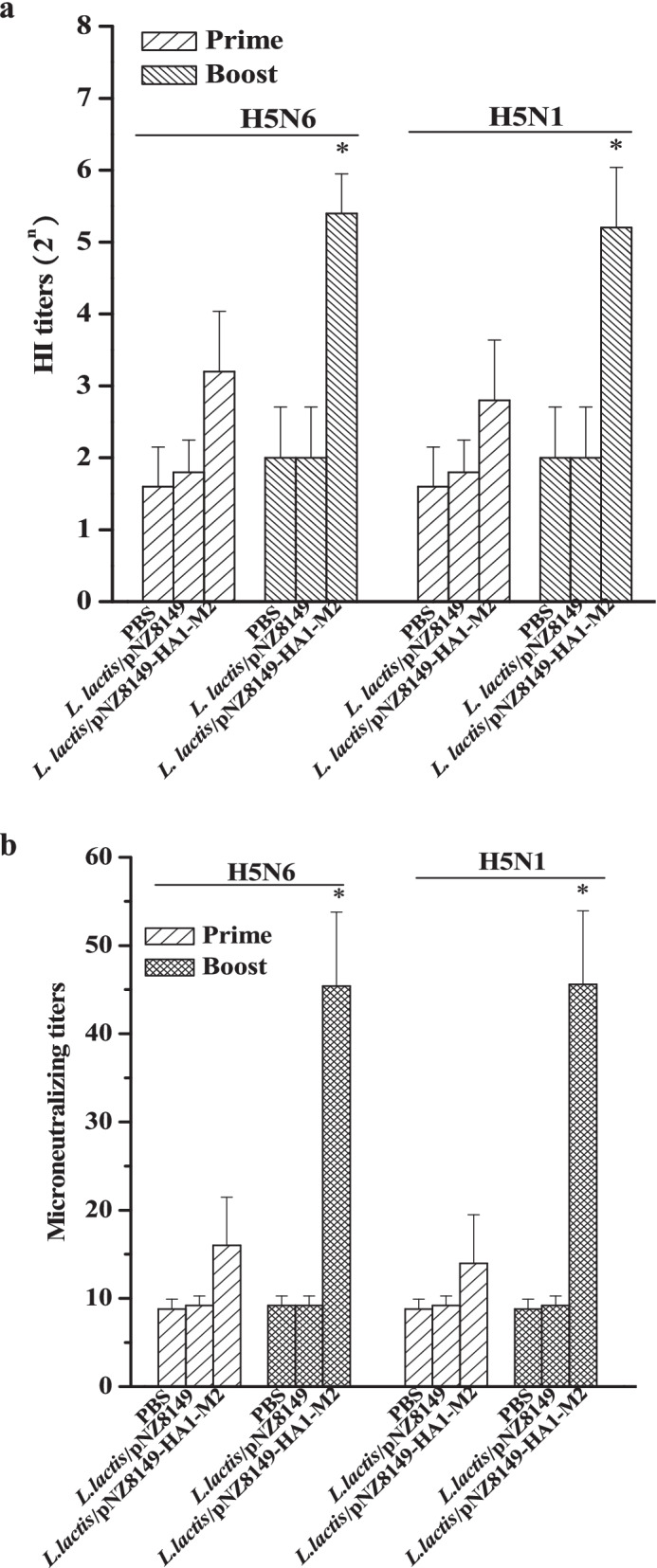
HI assay and microneutralization assay. **a** HI titers. A value less than 2^4^ was considered not statistically significant. **b** Microneutralization titers. A value less than 40 was considered not statistically significant. Data are presented as the means ± SD. Asterisks indicate significant differences compared to the PBS and *L. lactis*/pNZ8149 controls (*p* < 0.05)

The sera isolated from the vaccinated chickens were also analyzed by HI and microneutralization assays. As shown in Fig. 4a and b, there were no significant HI titers or neutralizing antibodies detected in any group after the prime immunization (at day 14). By contrast, the HI titers and neutralizing antibodies against H5N6 or H5N1 in the *L. lactis*/pNZ8149-HA1-M2 group were increased significantly after the boost immunization (at day 28). These data were consistent with IgG detection results described above, indicating that chickens orally vaccinated with *L. lactis*/pNZ8149-HA1-M2 after the prime-boost immunization could produce the highest levels of neutralizing antibodies against viral infection in vitro.

### Protection against H5Nx virus challenge

**Fig. 5:**
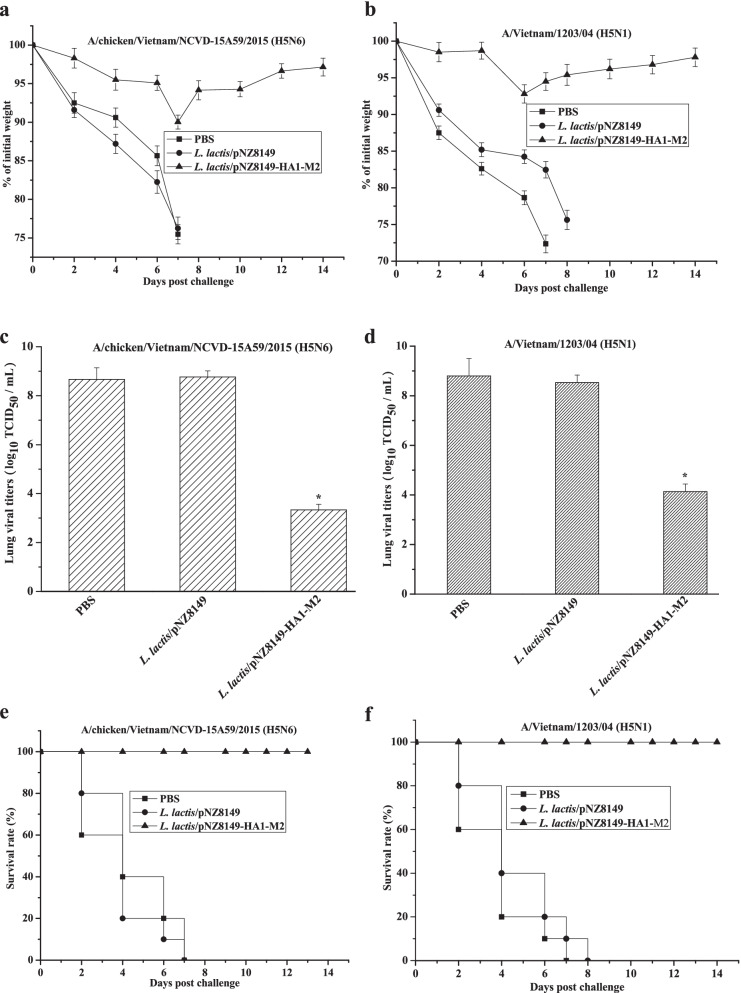
Protective immunity against lethal challenge with H5N6 or H5N1 virus. At day 30 after the first immunization, chickens were challenged intranasally with 20 μl of 5 × LD_50_ (lethal dose) of A/chicken/Vietnam/NCVD-15A59/2015 (H5N6) (**a**, **c** and **e**) or A/Vietnam/1203/04 (H5N1) (**b**, **d** and **f**) (*n* = 10 chickens / group). The data for lung virus titer (*n* = 5 chickens/ group) are presented as the means ± SD. Asterisks indicate significant differences compared to the PBS and *L. lactis*/pNZ8149 controls (*p* < 0.05)

Lastly, to assess protective immunity, all vaccinated chickens were transferred to the enhanced BSL-3 facilities and challenged with a lethal dosage of A/chicken/Vietnam/NCVD-15A59/2015 (H5N6) or A/Vietnam/1203/04 (H5N1) virus at day 30 after the first immunization. As shown in Fig. 5, the control groups that received PBS or *L. lactis*/pNZ8149 suffered severe body weight loss, high lung viral titers and died within 8 days after viral challenge. In contrast, chickens orally vaccinated with *L. lactis*/pNZ8149-HA1-M2 did not show significant weight loss, had lower lung viral titers, and survived 100% against H5N6 and H5N1, respectively. These results indicated that the oral administration of *L. lactis*/pNZ8149-HA1-M2 could confer protection against divergent influenza H5Nx viruses in the chicken model.

## Discussion

Current influenza vaccines for poultry based on surface antigens do not provide protection against different H5Nx viruses. Previous studies have shown that recombinant *L. lactis* could be designed as a vehicle to deliver influenza neuraminidases (NA) that induced strain-specific protection against H5N1 viral infection [21, 22]. In contrast to influenza HA and NA, M2 protein is highly conserved and serves as a promising influenza vaccine target. Therefore, M2 is chosen for generating *L. lactis* based vaccine, which may contribute to eliciting protective immunity against heterologous influenza H5Nx viruses. Furthermore, because these *L. lactis* expression plasmids contain antibiotic selection markers, they are associated with safety issues when administered via the intranasal or oral administration routes. Based on these findings, we hypothesized that the oral administration of recombinant *L. lactis* based vaccine could provide an alternative approach for influenza vaccine development that would be able to induce protection. The results of the present study demonstrated the potential of *L. lactis* based vaccine producing H5 HA1 and the highly conserved influenza M2 protein to contribute to protective immunity against challenge with homologous and heterologous influenza viruses. Therefore, the food-grade *L. lactis* based vaccine can be considered a promising candidate for influenza vaccine development.

It is generally accepted that orally administered *L. lactis* reaches the intestine and are taken up by the M cells of Peyer’s patches, where they are transported across the epithelium to the underlying antigen-presenting cells (APCs), primarily dendritic cells [26]. The antigens are then processed and appropriately presented by the APCs to elicit secretory IgA responses, which may prevent viral infection [26, 27]. In addition, *L. lactis*, as a vector, has immune-stimulating properties, which is an important advantage in the case of vaccines [27]. It is noteworthy that only a weak immune response is elicited against the *L. lactis* vector itself, while the major immune responses are directed primarily against the expressed heterologous antigens [26, 28]. This effect is a significant advantage since a strong immune response against vaccine carriers is known to diminish the response against heterologous antigens [28]. However, there is a scientific challenge regarding how to enhance the immunogenicity of recombinant *L. lactis* based vaccines via the oral administration route. A high level of antigen expression in vitro is required, since the gut residency of recombinant *L. lactis* harbouring heterologous antigen is rather transient in avian system [24]. Therefore, transient gene expression system, as a fast, flexible and reproducible approach, may be optimized for producing sufficient antigens that elicit the significantly adaptive immune responses. Furthermore, the use of mucosal adjuvants is supposed to be an effective way to induce strong immune responses. Another possibility is *L. lactis* based vaccines expressing antigen in cells so that antigens can escape the degradation of stomach acid and release in the intestine. This method is more scientific since it requires a small amount of a vaccine to achieve an ideal immune efficacy via the oral administration route. In this study, we investigated the immunogenicity of *L. lactis*/pNZ8149-HA1-M2 without the use of a mucosal adjuvant. Of note, it is critical to determine the optimal dosage of *L. lactis*/pNZ8149-HA1-M2 via the oral administration route. In practice, it was shown that oral vaccination at prime-boost immunization with *L. lactis*/pNZ8149-HA1-M2 was a good schedule to achieve the desired protection. Particularly, no clinical signs were observed in the vaccinated chickens based on this schedule.

Although the M2 protein is a weak immunogen and anti-M2 antibodies have no neutralizing activities [29], *L. lactis*/pNZ8149-HA1-M2 elicited strong humoral immune responses and neutralizing antibodies against H5N6 and H5N1 (Fig. 2a, b and Fig. 4b) since the HA1 protein was fused with M2. These results are consistent with previous studies demonstrating that anti-HA1 antibodies could neutralize the influenza virus [30]. Moreover, *L. lactis*/pNZ8149-HA1-M2 also induced a robust mucosal immune response (Fig. 2c, d), and secretory IgA production is helpful for viral clearance [26]. In further support of the immunogenicity of *L. lactis*/pNZ8149-HA1-M2, an enhanced level of the cellular immune response was observed, indicating that IFN-γ (Fig. 3), an effector of the Th1 immune response, is involved in protection from viral infection.

Viral challenge is a gold-standard way to evaluate the immune efficacy of a new influenza vaccine. An ideal influenza vaccine would be able to provide broad protection against divergent influenza viruses [31]. Particularly, the immune sera from *L. lactis*/pNZ8149-HA1-M2-immunized chickens played an important role in providing chickens protective immunity against viral infection, as observed by mild weight loss and low lung viral titers (Fig. 5). We speculated that protection could be related to the generation of anti-M2 and anti-HA1 antibodies, which greatly contribute to protective immunity, supporting our hypothesis that antibodies against HA1 have neutralizing activities and anti-M2 antibodies provide cross-reactivity. Thus, *L. lactis*/pNZ8149-HA1-M2 vaccine that is capable of inducing HA1-specific and M2-specific antibodies and protection against other influenza virus subtypes offers a new influenza vaccine candidate for further clinical evaluation.

In addition, it will be a challenge to produce a safe and effective vaccine if a pandemic suddenly emerges and rapidly spreads in poultry. Furthermore, another issue is that mutation of the HA1 subunit occurs frequently and results in antigenic drift or antigenic shifts that require a seasonal influenza vaccine that is updated annually. The platform developed in the present study based on a food-grade *L. lactis* expression system can completely address this issue and would be rapidly adjustable to facilitate the construction of a new influenza vaccine by replacing the HA1 subunit and matching the circulating strain. In this regard, *L. lactis*/pNZ8149-HA1-M2 is an alternative approach to develop an influenza vaccine for poultry that would quickly respond to H5Nx outbreaks.

## Conclusions

To sum up, the present study describes a potential approach for providing protective immunity against H5Nx viruses based on *L. lactis* expression system in a chicken model via oral administration without the use of a mucosal adjuvant. The results highlight that *L. lactis*/pNZ8149-HA1-M2 would be an effective vaccine candidate for an influenza H5Nx vaccine development in poultry.

## Methods

### Ethical statement

All experimental protocols involving animals were approved by the ethics committee of Southwest Jiaotong University. All animal procedures were carried out in **accordance with** the Guidelines for Use and Care of Experimental Animals in Southwest Jiaotong University. The study was carried out in compliance with the ARRIVE guidelines [32].

### Construction of recombinant *L. lactis* expressing HA1-M2

To generate the *L. lactis* based vaccine, the HA1 gene (987 bp) of A/chicken/Vietnam/NCVD-15A59/2015 (H5N6) (GenBank accession number: AY651334) was PCR amplified from pCDNA3.1-HA using the primers HA1-F (5′ CATGCCATGGATCAGATTTGCATTGGTT 3′) and HA1-R (5′ CCGCCGCCGCCGCGGCTCTTTTTCTTTTTC 3′), with the *Nco*I restriction site and GS linker sequence underlined, respectively. Similarly, the M2 gene of A/Vietnam/1203/2004 (H5N1) (GenBank: AAT70528, 291 bp) was PCR amplified from pGEM-M2 (Synthesized by Eurofins Technology Service Company, Suzou, China) using the primers M2-F (5′ GGCGGCGGCGGCGCCAGTCTTCTAACCGAG 3′) and M2-R (5′ CGGGGTACCTTACTCCAGCTCTAT 3′), with the GS linker sequence and restriction enzyme site *Kpn*I underlined, respectively. The HA1 and M2 genes were fused into HA1-M2 using the primers HA1-F and M-R through the GS linker. The resulting HA1-M2 (*Nco*I/*Kpn*I) fragment was subcloned intopNZ8149 (Fig. 1a). Recombinant pNZ8149-HA1-M2 was transformed into competent *L. lactis* NZ3900, which was then grown on lactose agar plates for food-grade selection at 30 °C for 2 days. Subsequently, a single positive clone was grown in 5 ml of M17 medium overnight at 30 °C without shaking. *L. lactis* containing the empty plasmid pNZ8149 (*L. lactis*/pNZ8149) was used as a negative control for subsequent analyses.

### Western blot analysis

The expression of recombinant *L. lactis*/pNZ8149-HA1-M2 was determined by Western blot analysis. Briefly, 5 × 10^5^ cells of *L. lactis*/pNZ8149-HA1-M2 cells were mixed with 60 μl of 6 × loading buffer and boiled for 10 min, after which the proteins were resolved by sodium dodecyl sulfate-polyacrylamide gel electrophoresis (SDS-PAGE) and transferred to nitrocellulose membranes (Bio-Rad, Hercules, California, USA). The membranes were blocked with 5% skim milk and then incubated with a 1:500-diluted monoclonal mouse anti-HA or anti-M2 antibody (kindly provided by Beiresources, Manassas, VA, USA) overnight at 4 °C. Affinity-purified horseradish peroxidase (HRP)-conjugated anti-mouse IgG (Sigma-Aldrich Corporation, St. Louis, MO, USA) was used as the secondary antibody. Finally, the membrane was visualized using enhanced chemiluminescence reagents (GE Healthcare) according to the manufacturer’s instructions. Western blot analysis was performed by three independent experiments.

### Immunofluorescence assay

Immunofluorescence assay was performed as described previously [22]. Briefly, a total of 5 × 10^5^ cells of recombinant *L. lactis*/pNZ8149-HA1-M2 were fixed with 4% paraformaldehyde. Monoclonal mouse anti-HA or anti-M2 antibody was used as primary antibody and goat anti-mouse IgG antibody conjugated with fluorescein isothiocyanate (FITC) (R&D Systems, USA) was served as secondary antibody. Lastly, the cells were detected using microscope (Leica, Wetzlar, Germany), with *L. lactis*/pNZ8149 cells used as a negative control.

### Quantification of HA1-M2 expressed in *L. lactis* by whole-cell ELISA

Quantification of *L. lactis*/pNZ8149-HA1-M2 expressing the HA1-M2 fusion protein was determined by ELISA as describe previously [24]. Briefly, The ELISA plates were coated with 100 μl of cell lysates of 1 × 10^8^ CFU *L. lactis*/pNZ8149-HA1-M2 overnight at 4 °C. Before and after every step, the plates were washed with PBS + 0.5% Tween 20. 100 μl of a monoclonal mouse anti-HA or anti-M2 antibody (0, 10, 20, 30, 40, 50, 60, 70, 80, 90 or 100 μg/ml) diluted to 1:500 in PBS containing 2% BSA was added to each well. After incubation at 37 °C for 2 h, 1 mg/ml horseradish peroxidase (HRP)-conjugated goat anti-mouse IgG antibody was added and incubated at room temperature for 1 h. Finally, 100 μl of 3,3′,5,5′-tetramethylbenzidine (TMB) was added and reacted for 25 min in the dark, and 100 μl of 2 mol/l H_2_SO_4_ was used to stop the reaction. The absorbance at 450 nm was measured using a microplate reader (BioTek, USA). *L. lactis*/pNZ8149 served as a negative control.

### Animal experiments and sample collection

*L. lactis*/pNZ8149 and *L. lactis*/pNZ8149-HA1-M2 cells were adjusted to 10^12^ colony-forming units (CFU)/ml with sterile PBS, respectively.

7-day-old white Leghorn chickens (SLC Laboratories, Shanghai, China) were housed as 5 chicks per cage (50 cm × 45 cm × 45 cm) in an environmentally controlled house. The chickens w**ere fed** a pathogen-free diet and water.

For animal experiments, the chickens (*n* = 50 chickens/group) were orally immunized with 500 μl of *L. lactis*/pNZ8149-HA1-M2. Prime immunization was performed on days 1 and 2, and boost immunization was scheduled on days 16 and 17. The same volume of PBS or *L. lactis*/pNZ8149 was used as a control.

Blood (*n* = 10 chickens/group), intestine washes (*n* = 5 chickens/group) and spleen samples (*n* = 5 chickens/group) from the sacrificed chickens using CO_2_ inhalation for 5 min were collected at days 14 and 28 post prime immunization.

### Determination of antibody responses by ELISA

Humoral and mucosal immune responses were analyzed by ELISA. Recombinant H5 HA protein was purchased from Harbin Veterinary Research Institute, Chinese Academy of Agricultural Sciences (Harbin, China). The M2e peptide (SLLTEVETPIRNEWGCR) was synthesized by Eurofins Technology Service Company (Suzou, China). Two-fold diluted sera and intestine washes were tested for IgG and IgA, respectively.

The ELISA plates (Costar, Corning, Inc., USA) were coated with 100 μl of HA protein or M2e peptide overnight at 4 °C. The plates were washed three times with PBS-Tween 0.05% and then blocked with 100 μl of PBS with 10% of fetal bovine serum (FBS) (PBS-FBS) for 2 h at 37 °C. After washing with PBS containing 0.05% Tween 20 (PBS-T), the plates were incubated with 100 μl of serum dilution or intestine washes for 2 h at 37 °C. The plates were washed with PBS-T and incubated with 100 μl/well of biotinylated goat anti-chicken IgG or IgA (*Jackson ImmunoResearch* Laboratories, Inc., USA) at 1:5000 in PBS-T containing 3% BSA. After washing with PBS-T, 100 μl of 1:1000 diluted streptavidin alkaline phosphatase (R&D Systems, USA) was added to each well. After washing with PBS-T, wells were developed by p-Nitrophenyl Phosphate (pNPP) substrate (R&D Systems, USA) for 25 min at room temperature in the dark. The reaction was stopped by the addition of 50 μl/well of 2 mol/L NaOH. Optical density (OD) was measured using an ELISA reader (BioTek, USA) at 405 nm with a reference filter of 630 nm. The IgG and IgA titers were determined as the lowest dilution with an OD greater than the mean OD of negative controls plus 2 standard deviations.

To further determine cellular immune response, IFN-γ-secreting cell spots were measured on Multiscreen 96-well plates (Millipore) coated with cytokine-specific capture antibodies as described previously [18]. Splenocytes (*n* = 5 chickens/group) were isolated from the vaccinated chickens at days 14 and 28 post prime immunization. Briefly, 10^6^ spleen cells per well were cultured with or without M2e peptide (10 μg/ml) as an antigenic stimulator. After 36 h of incubation, the number of IFN-γ-secreting cells was counted using an ImmunoSpot reader (Cellular Technology, USA). Less than 20 IFN-γ spots were considered a background level.

### Hemagglutination inhibition assay

Serum hemagglutination inhibition (HI) titer against homologous [A/chicken/Vietnam/NCVD-15A59/2015 (H5N6)] or heterosubtypic [A/Vietnam/1203/04 (H5N1)] virus was determined by a standard HI microtiter assay using 4 HA units of virus as described previously [33]. Briefly, serum samples were treated with a receptor-destroying enzyme (Denka Seiken, Japan), heat-inactivated at 56 °C for 30 min, and tested by HI assay with 0.5% chicken red blood cells. The HI titers are presented by the highest serum dilution capable of preventing hemagglutination. HI titers less than 2^4^ were considered not statistically significant.

### Microneutralization assay

Virus-neutralizing activities of immune sera were determined by a conventional plaque reduction assay as described previously [17]. For virus growth inhibition assay, approximately 80% confluent Madin–Darby canine kidney (MDCK) cells were infected with 35 μl 100 50% tissue infective doses (TCID_50_) of A/chicken/Vietnam/NCVD-15A59/2015 (H5N6) or A/Vietnam/1203/04 (H5N1) virus. The virus solution was washed out and replaced with DMEM containing immune sera of various dilutions. Culture media were harvested at day 2 after infection, and the viral yield was estimated by hemagglutination activity assay using chicken red blood cells. The neutralizing antibody titer less than 40 was considered no significance.

### Viral challenge

To investigate cross-protective immunity, the vaccinated chickens were challenged with 20 μl of 5× LD_50_ of A/chicken/Vietnam/NCVD-15A59/2015 (H5N6) or A/Vietnam/1203/04 (H5N1) virus at day 30 after the first immunization. Lungs (*n* = 5 chickens/group) were isolated from the infected chickens at day 3 post challenge. Lung viral titers were determined using a plaque assay in MDCK cells. The limit of virus detection was 50 plaque-forming units. The survival rate was recorded for 14 days. All the virus challenge experiments were performed in enhanced animal biosafety level - 3 (BSL - 3) facilities. Body weight loss of greater than 25% was used as the criterion for euthanasia. All the surviving chickens were euthanized using CO_2_ inhalation for 5 min at 14 days post-infection.

### Statistical analysis

All data were represented as the mean ± standard deviation (SD). Student’s *t-*test and analysis of variance (ANOVA) were used to determine the significant differences between two or multiple sets of experimental data, respectively. A *p* value less than 0.05 was considered statistically significant.

## Supplementary Information

**Additional file 1: Supplementary Figure 1.** Full-length Western blots. M: Western blot marker; Lane 1: Cell culture supernatants of *L. lactis*/pNZ8149-HA1-M2; Lane 2: Cell lysates of *L. lactis*/pNZ8149-HA1-M2 (approximately 45 kDa); Lane 3: Cell lysates of *L. lactis*/pNZ8149; Lane 4: Cell culture supernatants of *L. lactis*/pNZ8149.

## Data Availability

The datasets generated and/or analyzed during the current study are available from the corresponding author on reasonable request.

## Abbreviations

ANOVA: Analysis of variance
APCs: Antigen-presenting cells
BSA: Bovine serum albumin
BSL-3: Biosafety level – 3
CFU: Colony-forming units
ELISA: Enzyme-linked immunosorbent assay
FITC: Fluorescein isothiocyanate
HA1: Hemagglutinin-1
HI: Hemagglutinination inhibition
HRP: Horseradish peroxidase
GALT: Gut-associated lymphoid tissue
GIT: Gastrointestinal tract
*L. lactis*: *Lactococcus lactis*
M2e: Ectodomain of matrix protein 2
MDCK: Madin–Darby canine kidney
NA: Neuraminidase
RT: Room temperature
SDS-PAGE: Sodium dodecyl sulfate-polyacrylamide gel electrophoresis
TCID_50_: 50% tissue infective doses
