# Supplementary material for: Protective immunity induced by oral vaccination with a recombinant Lactococcus lactis vaccine against H5Nx in chickens
Source: BMC Vet Res. 2022 Jan 3;18:3. doi: 10.1186/s12917-021-03109-z (PMC8720943; doi:10.1186/s12917-021-03109-z)
Supplement: Supplementary file 1 — Additional file 1: Supplementary Figure 1. Full-length Western blots. M: Western blot marker; Lane 1: Cell culture supernatants of L. lactis/pNZ8149-HA1-M2; Lane 2: Cell lysates of L. lactis/pNZ8149-HA1-M2 (approximately 45 kDa); Lane 3: Cell lysates of L. lactis/pNZ8149; Lane 4: Cell culture supernatants of L. lactis/pNZ8149. [file 12917_2021_3109_MOESM1_ESM.docx]

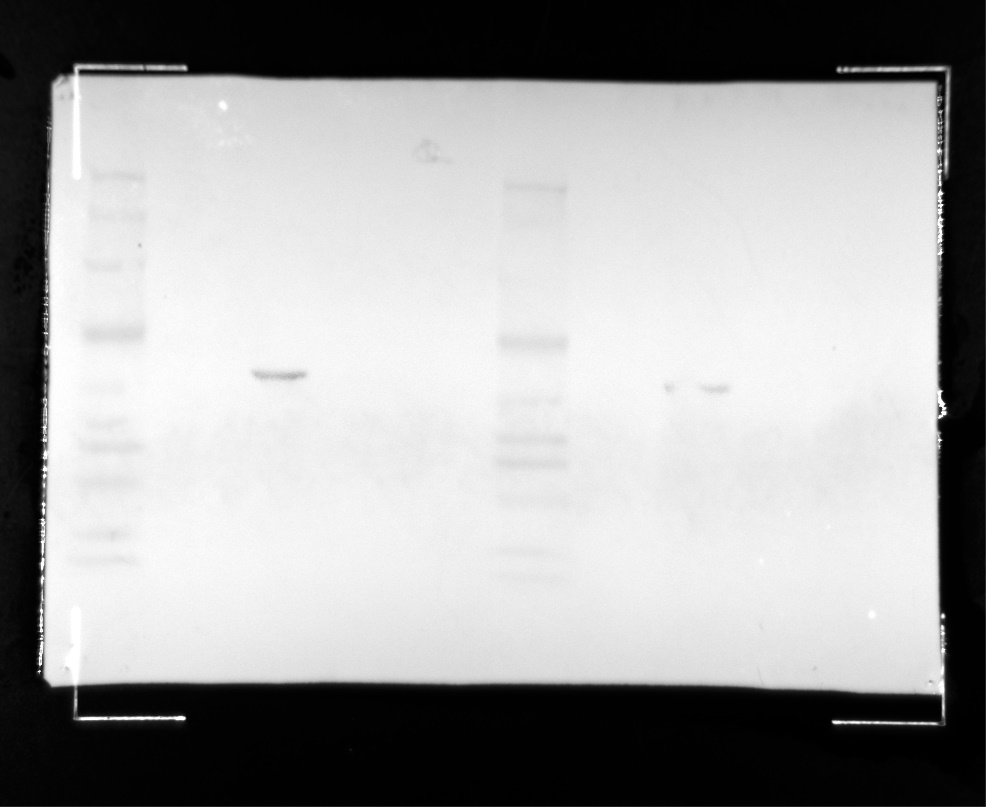


M 1 2 3 4 M 1 2 3 4

kDa

250

150

70

50

15

10

40

35

25

20

Supplementary Figure 1. Full-length Western blots. M: Western blot marker; Lane 1: Cell culture supernatants of *L. lactis*/pNZ8149-HA1-M2; Lane 2: Cell lysates of *L. lactis*/pNZ8149-HA1-M2 (approximately 45 kDa); Lane 3: Cell lysates of *L. lactis*/pNZ8149; Lane 4: Cell culture supernatants of *L. lactis*/pNZ8149.
